# Supplementary material for: OpenMM 7: Rapid development of high performance algorithms for molecular dynamics
Source: PLoS Comput Biol. 2017 Jul 26;13(7):e1005659. doi: 10.1371/journal.pcbi.1005659 (PMC5549999; doi:10.1371/journal.pcbi.1005659)
Supplement: S1 Source Code — (ZIP) [file pcbi.1005659.s002.zip › OpenMM-7.0.1-Source/docs/api-python/generated/simtk.openmm.app.modeller.Modeller.html]

Modeller — OpenMM 7.0.1.dev-5e86c4f documentation


# Modeller¶

*class* `simtk.openmm.app.modeller.``Modeller`(*topology*, *positions*)¶
:   Modeller provides tools for editing molecular models, such as adding water or missing hydrogens.

    To use it, create a Modeller object, specifying the initial Topology and atom positions. You can
    then call various methods to change the model in different ways. Each time you do, a new Topology
    and list of coordinates is created to represent the changed model. Finally, call getTopology()
    and getPositions() to get the results.

    `__init__`(*topology*, *positions*)¶
    :   Create a new Modeller object

        |  |  |
        | --- | --- |
        | Parameters: | - **topology** (*Topology*) – the initial Topology of the model - **positions** (*list*) – the initial atomic positions |

    Methods

    |  |  |
    | --- | --- |
    | `__init__`(topology, positions) | Create a new Modeller object |
    | `add`(addTopology, addPositions) | Add chains, residues, atoms, and bonds to the model. |
    | `addExtraParticles`(forcefield) | Add missing extra particles to the model that are required by a force field. |
    | `addHydrogens`([forcefield, pH, variants, ...]) | Add missing hydrogens to the model. |
    | `addSolvent`(forcefield[, model, boxSize, ...]) | Add solvent (both water and ions) to the model to fill a rectangular box. |
    | `convertWater`([model]) | Convert all water molecules to a different water model. |
    | `delete`(toDelete) | Delete chains, residues, atoms, and bonds from the model. |
    | `deleteWater`() | Delete all water molecules from the model. |
    | `getPositions`() | Get the atomic positions. |
    | `getTopology`() | Get the Topology of the model. |
    | `loadHydrogenDefinitions`(file) | Load an XML file containing definitions of hydrogens that should be added by addHydrogens(). |

    `getTopology`()¶
    :   Get the Topology of the model.

    `getPositions`()¶
    :   Get the atomic positions.

    `add`(*addTopology*, *addPositions*)¶
    :   Add chains, residues, atoms, and bonds to the model.

        Specify what to add by providing a new Topology object and the
        corresponding atomic positions. All chains, residues, atoms, and bonds
        contained in the Topology are added to the model.

        |  |  |
        | --- | --- |
        | Parameters: | - **addTopology** (*Topology*) – a Topology whose contents should be added to the model - **addPositions** (*list*) – the positions of the atoms to add |

    `delete`(*toDelete*)¶
    :   Delete chains, residues, atoms, and bonds from the model.

        You can specify objects to delete at any granularity: atoms, residues, or chains. Passing
        in an Atom object causes that Atom to be deleted. Passing in a Residue object causes that
        Residue and all Atoms it contains to be deleted. Passing in a Chain object causes that
        Chain and all Residues and Atoms it contains to be deleted.

        In all cases, when an Atom is deleted, any bonds it participates in are also deleted.
        You also can specify a bond (as a tuple of Atom objects) to delete just that bond without
        deleting the Atoms it connects.

        |  |  |
        | --- | --- |
        | Parameters: | **toDelete** (*list*) – a list of Atoms, Residues, Chains, and bonds (specified as tuples of Atoms) to delete |

    `deleteWater`()¶
    :   Delete all water molecules from the model.

    `convertWater`(*model='tip3p'*)¶
    :   Convert all water molecules to a different water model.

        @deprecated Use addExtraParticles() instead. It performs the same
        function but in a more general way.

        |  |  |
        | --- | --- |
        | Parameters: | **model** (*string=’tip3p’*) – the water model to convert to. Supported values are ‘tip3p’, ‘spce’, ‘tip4pew’, and ‘tip5p’. |

    `addSolvent`(*forcefield*, *model='tip3p'*, *boxSize=None*, *boxVectors=None*, *padding=None*, *numAdded=None*, *positiveIon='Na+'*, *negativeIon='Cl-'*, *ionicStrength=Quantity(value=0*, *unit=molar)*, *neutralize=True*)¶
    :   Add solvent (both water and ions) to the model to fill a rectangular box.

        The algorithm works as follows:

        1. Water molecules are added to fill the box.
        2. Water molecules are removed if their distance to any solute atom is less than the sum of their van der Waals radii.
        3. If the solute is charged and neutralize=True, enough positive or negative ions are added to neutralize it. Each ion is added by
           randomly selecting a water molecule and replacing it with the ion.
        4. Ion pairs are added to give the requested total ionic strength. Note that only monovalent ions are currently supported.

        The box size can be specified in any of several ways:

        1. You can explicitly give the vectors defining the periodic box to use.
        2. Alternatively, for a rectangular box you can simply give the dimensions of the unit cell.
        3. You can give a padding distance. The largest dimension of the solute (along the x, y, or z axis) is determined, and a cubic
           box of size (largest dimension)+2\*padding is used.
        4. You can specify the total number of molecules (both waters and ions) to add. A cubic box is then created whose size is
           just large enough hold the specified amount of solvent.
        5. Finally, if none of the above options is specified, the existing Topology’s box vectors are used.

        |  |  |
        | --- | --- |
        | Parameters: | - **forcefield** (*ForceField*) – the ForceField to use for determining van der Waals radii and atomic charges - **model** (*str=’tip3p’*) – the water model to use. Supported values are ‘tip3p’, ‘spce’, ‘tip4pew’, and ‘tip5p’. - **boxSize** (*Vec3=None*) – the size of the box to fill with water - **boxVectors** (*tuple of Vec3=None*) – the vectors defining the periodic box to fill with water - **padding** (*distance=None*) – the padding distance to use - **numAdded** (*int=None*) – the total number of molecules (waters and ions) to add - **positiveIon** (*string=’Na+’*) – the type of positive ion to add. Allowed values are ‘Cs+’, ‘K+’, ‘Li+’, ‘Na+’, and ‘Rb+’ - **negativeIon** (*string=’Cl-‘*) – the type of negative ion to add. Allowed values are ‘Cl-‘, ‘Br-‘, ‘F-‘, and ‘I-‘. Be aware   that not all force fields support all ion types. - **ionicStrength** (*concentration=0\*molar*) – the total concentration of ions (both positive and negative) to add. This   does not include ions that are added to neutralize the system.   Note that only monovalent ions are currently supported. - **neutralize** (*bool=True*) – whether to add ions to neutralize the system |

    *static* `loadHydrogenDefinitions`(*file*)¶
    :   Load an XML file containing definitions of hydrogens that should be added by addHydrogens().

        The built in hydrogens.xml file containing definitions for standard amino acids and nucleotides is loaded automatically.
        This method can be used to load additional definitions for other residue types. They will then be used in subsequent
        calls to addHydrogens().

    `addHydrogens`(*forcefield=None*, *pH=7.0*, *variants=None*, *platform=None*)¶
    :   Add missing hydrogens to the model.

        Some residues can exist in multiple forms depending on the pH and properties of the local environment. These
        variants differ in the presence or absence of particular hydrogens. In particular, the following variants
        are supported:

        Aspartic acid:
        :   ASH: Neutral form with a hydrogen on one of the delta oxygens
            ASP: Negatively charged form without a hydrogen on either delta oxygen

        Cysteine:
        :   CYS: Neutral form with a hydrogen on the sulfur
            CYX: No hydrogen on the sulfur (either negatively charged, or part of a disulfide bond)

        Glutamic acid:
        :   GLH: Neutral form with a hydrogen on one of the epsilon oxygens
            GLU: Negatively charged form without a hydrogen on either epsilon oxygen

        Histidine:
        :   HID: Neutral form with a hydrogen on the ND1 atom
            HIE: Neutral form with a hydrogen on the NE2 atom
            HIP: Positively charged form with hydrogens on both ND1 and NE2
            HIN: Negatively charged form without a hydrogen on either ND1 or NE2

        Lysine:
        :   LYN: Neutral form with two hydrogens on the zeta nitrogen
            LYS: Positively charged form with three hydrogens on the zeta nitrogen

        The variant to use for each residue is determined by the following rules:

        1. The most common variant at the specified pH is selected.
        2. Any Cysteine that participates in a disulfide bond uses the CYX variant regardless of pH.
        3. For a neutral Histidine residue, the HID or HIE variant is selected based on which one forms a better hydrogen bond.

        You can override these rules by explicitly specifying a variant for any residue. To do that, provide a list for the
        ‘variants’ parameter, and set the corresponding element to the name of the variant to use.

        A special case is when the model already contains a hydrogen that should not be present in the desired variant.
        If you explicitly specify a variant using the ‘variants’ parameter, the residue will be modified to match the
        desired variant, removing hydrogens if necessary. On the other hand, for residues whose variant is selected
        automatically, this function will only add hydrogens. It will never remove ones that are already present in the
        model, regardless of the specified pH.

        Definitions for standard amino acids and nucleotides are built in. You can call loadHydrogenDefinitions() to load
        additional definitions for other residue types.

        |  |  |
        | --- | --- |
        | Parameters: | - **forcefield** (*ForceField=None*) – the ForceField to use for determining the positions of hydrogens.   If this is None, positions will be picked which are generally   reasonable but not optimized for any particular ForceField. - **pH** (*float=7.0*) – the pH based on which to select variants - **variants** (*list=None*) – an optional list of variants to use. If this is specified, its   length must equal the number of residues in the model. variants[i]   is the name of the variant to use for residue i (indexed starting at   0). If an element is None, the standard rules will be followed to   select a variant for that residue. - **platform** (*Platform=None*) – the Platform to use when computing the hydrogen atom positions. If   this is None, the default Platform will be used. |
        | Returns: | a list of what variant was actually selected for each residue, in the same format as the variants parameter |
        | Return type: | list |

    `__delattr__`¶
    :   x.\_\_delattr\_\_(‘name’) <==> del x.name

    `__format__`()¶
    :   default object formatter

    `__getattribute__`¶
    :   x.\_\_getattribute\_\_(‘name’) <==> x.name

    `__hash__`¶

    `__reduce__`()¶
    :   helper for pickle

    `__reduce_ex__`()¶
    :   helper for pickle

    `__repr__`¶

    `__setattr__`¶
    :   x.\_\_setattr\_\_(‘name’, value) <==> x.name = value

    `__sizeof__`() → int¶
    :   size of object in memory, in bytes

    `__str__`¶

    `addExtraParticles`(*forcefield*)¶
    :   Add missing extra particles to the model that are required by a force
        field.

        Some force fields use “extra particles” that do not represent
        actual atoms, but still need to be included in the System. Examples
        include lone pairs, Drude particles, and the virtual sites used in some
        water models to adjust the charge distribution. Extra particles can be
        recognized by the fact that their element is None.

        This method is primarily used to add extra particles, but it can also
        remove them. It tries to match every residue in the Topology to a
        template in the force field. If there is no match, it will both add
        and remove extra particles as necessary to make it match.

        |  |  |
        | --- | --- |
        | Parameters: | **forcefield** (*ForceField*) – the ForceField defining what extra particles should be present |

High performance molecular simulation on GPUs


### Navigation

- Application Layer
  - Loaders and Setup
  - Representation and Manipulation
  - Simulation
  - Reporting Output
  - Extras
- Library Layer

©2015, Stanford University and the Authors.
|
Powered by Sphinx 1.3.1
& Alabaster 0.7.7
|
Page source
